# The genome sequence of the Thistle Conch moth, *Aethes cnicana *(Westwood, 1854)

**DOI:** 10.12688/wellcomeopenres.24252.1

**Published:** 2025-06-02

**Authors:** Mark R Young, Wellcome Sanger Institute Tree of Life Management, Samples and Laboratory team, Wellcome Sanger Institute Scientific Operations: Sequencing Operations, Wellcome Sanger Institute Tree of Life Core Informatics team, Tree of Life Core Informatics collective, Darwin Tree of Life Consortium

**Affiliations:** 1 University of Aberdeen School of Biological Sciences, Aberdeen, Scotland, UK

**Keywords:** Aethes cnicana, Thistle Conch, genome sequence, chromosomal, Lepidoptera

## Abstract

We present a genome assembly from a male specimen of *Aethes cnicana* (Thistle Conch; Arthropoda; Insecta; Lepidoptera; Tortricidae). The genome sequence has a total length of 412.26 megabases. Most of the assembly (96.09%) is scaffolded into 30 chromosomal pseudomolecules, including the Z sex chromosome. The mitochondrial genome has also been assembled, with a length of 15.66 kilobases.

## Species taxonomy

Eukaryota; Opisthokonta; Metazoa; Eumetazoa; Bilateria; Protostomia; Ecdysozoa; Panarthropoda; Arthropoda; Mandibulata; Pancrustacea; Hexapoda; Insecta; Dicondylia; Pterygota; Neoptera; Endopterygota; Amphiesmenoptera; Lepidoptera; Glossata; Neolepidoptera; Heteroneura; Ditrysia; Apoditrysia; Tortricoidea; Tortricidae; Tortricinae; Cochylini; *Aethes*; *Aethes cnicana* (Westwood, 1854) (NCBI:txid753148)

## Background

**Figure 1.**
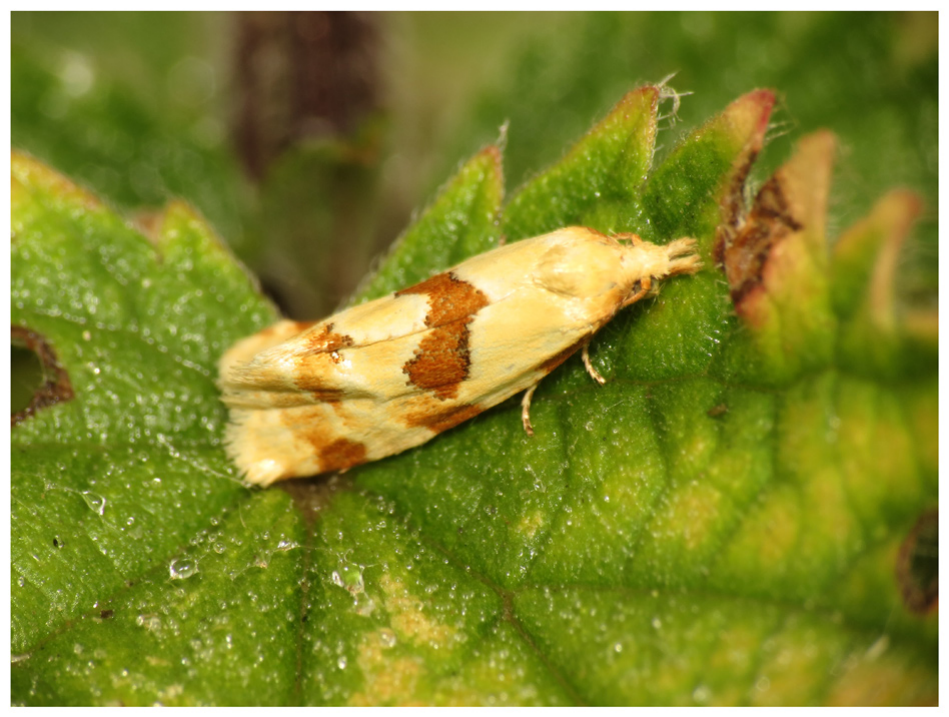
Photograph of *Aethes cnicana* by Donald Hobern (not the specimen used for genome sequencing).

*Aethes cnicana* (Westwood, 1854) is a distinctive moth in the family Tortricidae, with a wingspan of around 14–17 mm. It has dull creamy-yellow wings with an oblique orangey-brown median fascia, sometimes interrupted near the costa, and various other brown markings ( Figure 1). It resembles other members of its genus, but is most closely similar to *Aethes rubigana* (Treitschke, 1830), which is slightly larger (wingspan 16–19 mm). In *A. rubigana*, the median fascia is more often interrupted near the costa and has a curved outer margin, whereas in *A. cnicana* this margin is generally straight ( Bland *et al.*, 2015).

*Aethes cnicana* has a long emergence period but is most frequently recorded from late May to July or early August. It is generally found close to its foodplants, which are various species of thistle, including *Cirsium vulgare*. The eggs are laid on a flower or flower bud, and the larvae, which are yellowish or whitish with a slight green tinge, feed initially in the seeds before boring down into the pith of the stem. They hibernate fully fed from October to April, then pupate in the dead stem ( Bland *et al.*, 2015). In contrast, *Aethes rubigana* larvae feed on burdock ( *Arctium* spp.). Larvae of *Epiblema scutulana* (Denis & Schiffermüller, 1775) also develop in thistle stems and may be encountered alongside *A. cnicana*, but can be distinguished by their rose-pink colour.

This species is commonest in cool areas of Europe, especially the British and Irish Isles and Scandinavia, but extending down into central Europe. It avoids warmer southern European localities, but has scattered occurrences across eastern Europe and into Asia. It is not considered to be threatened, but the loss of marginal and open areas, with abundant thistles, has reduced its overall abundance.

The genome of the Thistle Conch, *Aethes cnicana*, was sequenced as part of the Darwin Tree of Life Project, a collaborative effort to sequence all named eukaryotic species in the Atlantic Archipelago of Britain and Ireland.

## Genome sequence report

### Sequencing data

The genome of a specimen of *Aethes cnicana* was sequenced using Pacific Biosciences single-molecule HiFi long reads, generating 46.40 Gb from 4.09 million reads, which were used to assemble the genome. GenomeScope analysis estimated the haploid genome size at 341.60 Mb, with a heterozygosity of 1.34% and repeat content of 34.57%. These estimates guided expectations for the assembly. Based on the estimated genome size, the sequencing data provided approximately 123 coverage. Hi-C sequencing produced 127.77 Gb from 846.19 million reads, and was used to scaffold the assembly. Table 1 summarises the specimen and sequencing details.

**Table 1.** Specimen and sequencing data for *Aethes cnicana*.

Project information			
**Study title**	Aethes cnicana (thistle conch)		
**BioProject**	PRJEB68256		
**Species**	*Aethes cnicana*		
**BioSpecimen**	SAMEA112198525		
**NCBI taxonomy ID**	753148		
Specimen information			
**Technology**	**ToLID**	**BioSample accession**	**Organism part**
**PacBio long read sequencing**	ilAetCnic1	SAMEA112198547	Head and thorax
**Hi-C sequencing**	ilAetCnic1	SAMEA112198547	Head and thorax
Sequencing information			
**Platform**	**Run accession**	**Read count**	**Base count (Gb)**
**Hi-C Illumina NovaSeq 6000**	ERR12259814	8.46e+08	127.77
**PacBio Revio**	ERR12257390	4.09e+06	46.4

### Assembly statistics

The primary assembly was generated, and contigs corresponding to an alternate haplotype were also deposited in INSDC databases. The assembly was improved by manual curation, which corrected 557 misjoins or missing joins and removed 53 haplotypic duplications. These interventions decreased the scaffold count by 26.32% and increased the scaffold N50 by 13.59%. The final assembly has a total length of 412.26 Mb in 769 scaffolds, with 2,832 gaps, and a scaffold N50 of 13.71 Mb ( Table 2).

**Table 2.** Genome assembly data for *Aethes cnicana*.

Genome assembly		
Assembly name	ilAetCnic1.1	
Assembly accession	GCA_963971205.1	
*Alternate haplotype accession*	*GCA_963971215.1*	
Assembly level for primary assembly	chromosome	
Span (Mb)	412.26	
Number of contigs	3,601	
Number of scaffolds	769	
Longest scaffold (Mb)	30.42	
**Assembly metric**	**Measure**	*Benchmark*
Contig N50 length	0.22 Mb	*≥ 1 Mb*
Scaffold N50 length	13.71 Mb	*= chromosome N50*
Consensus quality (QV)	Primary: 59.2; alternate: 59.4; combined: 59.3	*≥ 40*
*k*-mer completeness	Primary: 77.92%; alternate: 77.79%; combined: 99.18%	*≥ 95%*
BUSCO *	C:90.0%[S:88.9%,D:1.1%], F:1.8%,M:8.2%,n:5,286	*S > 90%; D < 5%*
Percentage of assembly mapped to chromosomes	96.09%	*≥ 90%*
Sex chromosomes	Z	*localised homologous pairs*
Organelles	Mitochondrial genome: 15.66 kb	*complete single alleles*

* BUSCO scores based on the lepidoptera_odb10 BUSCO set using version 5.5.0. C = complete [S = single copy, D = duplicated], F = fragmented, M = missing, n = number of orthologues in comparison.

**Figure 2.**
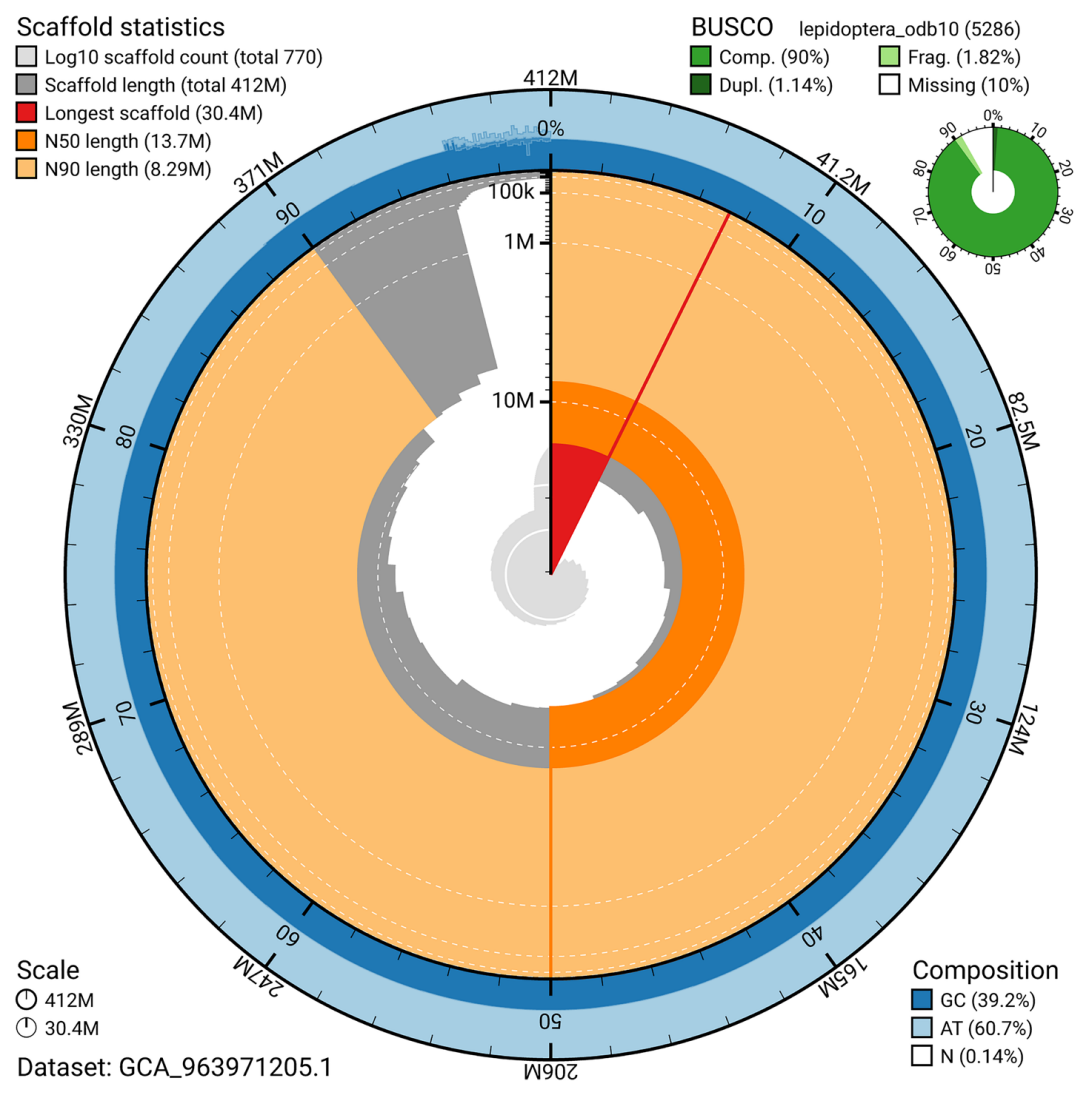
Genome assembly of *Aethes cnicana*, ilAetCnic1.1: metrics. The BlobToolKit snail plot provides an overview of assembly metrics and BUSCO gene completeness. The circumference represents the length of the whole genome sequence, and the main plot is divided into 1,000 bins around the circumference. The outermost blue tracks display the distribution of GC, AT, and N percentages across the bins. Scaffolds are arranged clockwise from longest to shortest and are depicted in dark grey. The longest scaffold is indicated by the red arc, and the deeper orange and pale orange arcs represent the N50 and N90 lengths. A light grey spiral at the centre shows the cumulative scaffold count on a logarithmic scale. A summary of complete, fragmented, duplicated, and missing BUSCO genes in the lepidoptera_odb10 set is presented at the top right. An interactive version of this figure is available at https://blobtoolkit.genomehubs.org/view/GCA_963971205.1/dataset/GCA_963971205.1/snail.

**Figure 3.**
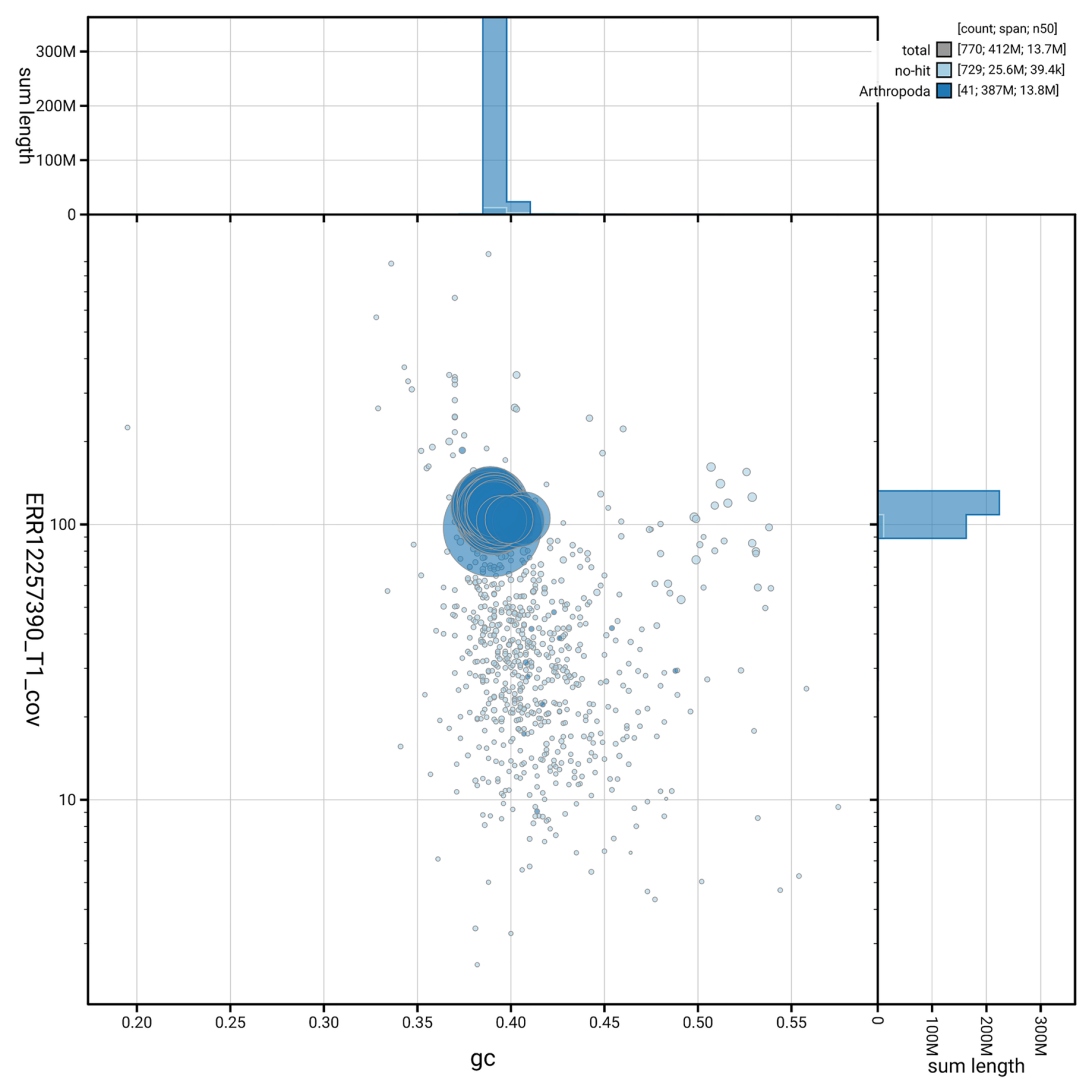
Genome assembly of *Aethes cnicana*, ilAetCnic1.1: BlobToolKit GC-coverage plot. Blob plot showing sequence coverage (vertical axis) and GC content (horizontal axis). The circles represent scaffolds, with the size proportional to scaffold length and the colour representing phylum membership. The histograms along the axes display the total length of sequences distributed across different levels of coverage and GC content. An interactive version of this figure is available at https://blobtoolkit.genomehubs.org/view/GCA_963971205.1/dataset/GCA_963971205.1/blob.

**Figure 4.**
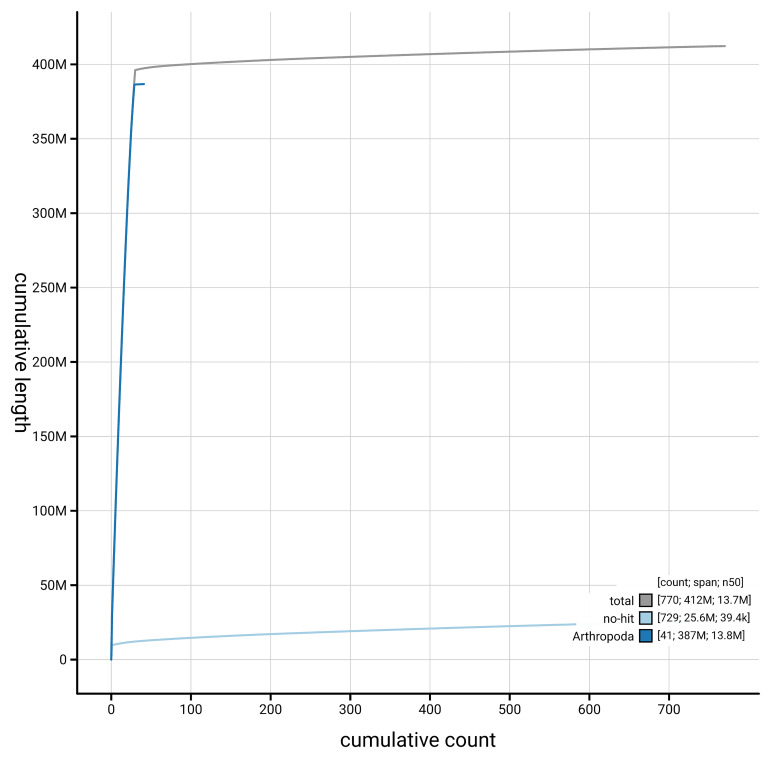
Genome assembly of *Aethes cnicana,* ilAetCnic1.1: BlobToolKit cumulative sequence plot. The grey line shows cumulative length for all scaffolds. Coloured lines show cumulative lengths of scaffolds assigned to each phylum using the buscogenes taxrule. An interactive version of this figure is available at https://blobtoolkit.genomehubs.org/view/GCA_963971205.1/dataset/GCA_963971205.1/cumulative.

The snail plot in Figure 2 provides a summary of the assembly statistics, indicating the distribution of scaffold lengths and other assembly metrics. Figure 3 shows the distribution of scaffolds by GC proportion and coverage. Figure 4 presents a cumulative assembly plot, with separate curves representing different scaffold subsets assigned to various phyla, illustrating the completeness of the assembly.

**Figure 5.**
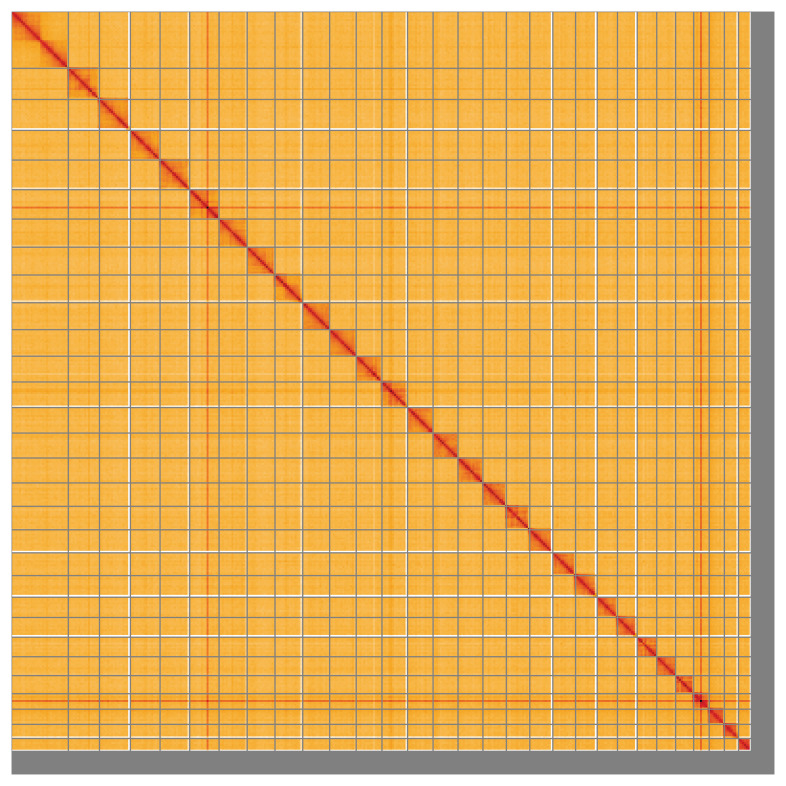
Hi-C contact map of the ilAetCnic1.1 assembly. The map was generated using PretextSnapshot. Chromosomes are shown in order of size and labelled with chromosome accession numbers (left) and names (top). An interactive version of this figure in HiGlass may be viewed at https://genome-note-higlass.tol.sanger.ac.uk/l/?d=Yl2RquPpTmCThqfTp0tBTQ.

Most of the assembly sequence (96.09%) was assigned to 30 chromosomal-level scaffolds, representing 29 autosomes and the Z sex chromosome. These chromosome-level scaffolds, confirmed by Hi-C data, are named according to size ( Figure 5; Table 3). During curation, the Z chromosome was identified based on synteny with the genome of *Pandemis heparana* (GCA_963854515.1).

**Table 3.** Chromosomal pseudomolecules in the genome assembly of *Aethes cnicana*, ilAetCnic1.

INSDC accession	Name	Length (Mb)	GC%
OZ020273.1	1	16.67	39
OZ020274.1	2	16.55	39
OZ020275.1	3	15.88	39
OZ020276.1	4	15.87	38.5
OZ020277.1	5	15.73	39
OZ020278.1	6	14.84	39
OZ020279.1	7	15.06	39
OZ020280.1	8	14.89	39
OZ020281.1	9	14.42	39
OZ020282.1	10	14.23	39
OZ020283.1	11	13.79	38.5
OZ020284.1	12	13.71	39
OZ020285.1	13	13.65	39
OZ020286.1	14	13.35	39
OZ020287.1	15	13.34	39
OZ020288.1	16	12.55	39.5
OZ020289.1	17	12.53	39
OZ020290.1	18	12.35	39
OZ020291.1	19	12.21	39
OZ020292.1	20	11.57	39
OZ020293.1	21	10.86	39.5
OZ020294.1	22	10.55	39
OZ020295.1	23	10.45	39
OZ020296.1	24	10.11	39
OZ020297.1	25	9.69	39.5
OZ020298.1	26	8.29	40.5
OZ020299.1	27	8.13	40.5
OZ020300.1	28	7.58	39.5
OZ020301.1	29	6.85	40
OZ020272.1	Z	30.42	39
OZ020302.1	MT	0.02	19.5

The mitochondrial genome was also assembled. This sequence is included as a contig in the multifasta file of the genome submission and as a standalone record.

### Assembly quality metrics

The estimated Quality Value (QV) and *k*-mer completeness metrics, along with BUSCO completeness scores, were calculated for each haplotype and the combined assembly. The QV reflects the base-level accuracy of the assembly, while *k*-mer completeness indicates the proportion of expected *k*-mers identified in the assembly. BUSCO scores provide a measure of completeness based on benchmarking universal single-copy orthologues.

The combined primary and alternate assemblies achieve an estimated QV of 59.3. The *k*-mer recovery for the primary haplotype is 77.92%, and for the alternate haplotype 77.79%; the combined primary and alternate assemblies have a *k*-mer recovery of 99.18%. BUSCO v.5.5.0 analysis using the lepidoptera_odb10 reference set ( *n* = 5,286) identified 90.0% of the expected gene set (single = 88.9%, duplicated = 1.1%).

Table 2 provides assembly metric benchmarks adapted from Rhie *et al.* (2021) and the Earth BioGenome Project Report on Assembly Standards September 2024. The assembly achieves the EBP reference standard of **5.C.Q59**.

## Methods

### Sample acquisition

The specimen used for genome sequencing was an adult male *Aethes cnicana* (specimen ID SAN00002596, ToLID ilAetCnic1), collected from Scougal Moor, Isle of Bute, Argyll and Bute, Scotland, United Kingdom (latitude 55.778, longitude –5.047) on 2022-07-02. It was netted whilst in flight near its foodplant. The specimen was collected and identified by Mark R. Young (University of Aberdeen) and preserved on dry ice. Metadata collection for samples adhered to the Darwin Tree of Life project standards described by Lawniczak *et al*. (2022).

### DNA extraction

The workflow for high molecular weight (HMW) DNA extraction at the Wellcome Sanger Institute (WSI) Tree of Life Core Laboratory includes a sequence of procedures: sample preparation and homogenisation, DNA extraction, fragmentation and purification. Detailed protocols are available on protocols.io ( Denton *et al.*, 2023b). The ilAetCnic1 sample was prepared for DNA extraction by weighing and dissecting it on dry ice ( Jay *et al.*, 2023). Tissue from the head and thorax was homogenised using a PowerMasher II tissue disruptor ( Denton *et al.*, 2023a). HMW DNA was extracted in the WSI Scientific Operations core using the Automated MagAttract v2 protocol ( Oatley *et al.*, 2023a). For ultra-low input (ULI) PacBio sequencing, DNA was fragmented using the Covaris g-TUBE method ( Oatley *et al.*, 2023b). Sheared DNA was purified by solid-phase reversible immobilisation, using AMPure PB beads to eliminate shorter fragments and concentrate the DNA ( Strickland *et al.*, 2023). The concentration of the sheared and purified DNA was assessed using a Nanodrop spectrophotometer and Qubit Fluorometer using the Qubit dsDNA High Sensitivity Assay kit. Fragment size distribution was evaluated by running the sample on the FemtoPulse system.

### PacBio library preparation and sequencing

Library preparation and sequencing were performed at the WSI Scientific Operations core. The sample was sheared to approximately 10 kb using a Covaris g-TUBE prior to library preparation. Ultra-low input libraries were prepared using the PacBio SMRTbell® Express Template Prep Kit 2.0 and the SMRTbell® gDNA Sample Amplification Kit (Pacific Biosciences, California, USA). DNA was normalised to 20 ng. Initial processing steps – including removal of single-stranded overhangs, DNA damage repair, and end repair/A-tailing – were performed according to the manufacturer’s instructions. Amplification adapters from the SMRTbell® gDNA Sample Amplification Kit were then ligated, followed by a 0.85X pre-PCR clean-up using Promega ProNex beads. The sample was divided into two aliquots for dual PCR reactions (A and B), which were carried out according to the manufacturer’s protocol. A 0.85X post-PCR clean-up was performed for each reaction using ProNex beads. DNA concentration was measured using a Qubit Fluorometer v4.0 (Thermo Fisher Scientific) and the Qubit HS Assay Kit. Fragment size was analysed using the Agilent Femto Pulse Automated Pulsed Field CE Instrument (Agilent Technologies) and the gDNA 55 kb BAC analysis kit.

PCR reactions A and B were then pooled, ensuring a combined DNA mass of at least 500 ng in 47.4 μL. The pooled sample underwent a second round of DNA damage repair, end repair/A-tailing, and ligation of additional hairpin adapters. A 1X clean-up was performed with ProNex beads. DNA concentration and fragment size were reassessed using the Qubit and Agilent Femto Pulse instruments. Size selection was performed using the PippinHT system (Sage Science), with target fragment size determined from Femto Pulse analysis, typically between 4,000 and 9,000 bp. Size-selected libraries were cleaned using 1.0X ProNex beads and normalised to 2 nM prior to sequencing.

Samples were sequenced on a Revio instrument (Pacific Biosciences, California, USA). Prepared libraries were normalised to 2 nM, and 15 μL was used for making complexes. Primers were annealed and polymerases were bound to create circularised complexes according to manufacturer’s instructions. The complexes were purified with the 1.2X clean up with SMRTbell beads. The purified complexes were then diluted to the Revio loading concentration (in the range 200–300 pM), and spiked with a Revio sequencing internal control. Samples were sequenced on Revio 25M SMRT cells (Pacific Biosciences, California, USA). The SMRT link software, a PacBio web-based end-to-end workflow manager, was used to set-up and monitor the run, as well as perform primary and secondary analysis of the data upon completion.

### Hi-C crosslinking, library preparation and sequencing

Hi-C data were generated from 20–50 mg of frozen head and thorax tissue (stored at –80 °C). from the ilAetCnic1 sample using the Arima-HiC v2 kit (Arima Genomics). As per manufacturer’s instructions, tissue was fixed, and the DNA crosslinked using a TC buffer with 22% formaldehyde concentration, and a final formaldehyde concentration of 2%. The tissue was then homogenised using the Diagnocine Power Masher-II. The crosslinked DNA was digested using a restriction enzyme master mix, then biotinylated and ligated. A clean up was performed with SPRIselect beads prior to library preparation. DNA concentration was quantified using the Qubit Fluorometer v4.0 (Thermo Fisher Scientific) and Qubit HS Assay Kit, and sample biotinylation percentage was estimated using the Arima-HiC v2 QC beads.

For Hi-C library preparation, the biotinylated DNA constructs were fragmented using a Covaris E220 sonicator and size-selected to 400–600 bp using SPRISelect beads. DNA was then enriched using Arima-HiC v2 Enrichment beads. The NEBNext Ultra II DNA Library Prep Kit (New England Biolabs) was used for end repair, A-tailing, and adapter ligation, following a modified protocol in which library preparation is carried out while the DNA remains bound to the enrichment beads. PCR amplification was performed using KAPA HiFi HotStart mix and custom dual-indexed adapters (Integrated DNA Technologies) in a 96-well plate format. Depending on sample concentration and biotinylation percentage determined at the crosslinking stage, samples were amplified for 10–16 PCR cycles. Post-PCR clean-up was carried out using SPRISelect beads. The libraries were quantified using the Accuclear Ultra High Sensitivity dsDNA Standards Assay kit (Biotium) and normalised to 10 ng/μL before sequencing. Hi-C sequencing was performed on the Illumina NovaSeq 6000 instrument using 150 bp paired-end reads.

### Genome assembly, curation and evaluation

***Assembly***

Prior to assembly of the PacBio HiFi reads, a database of *k*-mer counts ( *k* = 31) was generated from the filtered reads using FastK. GenomeScope2 ( Ranallo-Benavidez *et al.*, 2020) was used to analyse the *k*-mer frequency distributions, providing estimates of genome size, heterozygosity, and repeat content.

The HiFi reads were first assembled using Hifiasm ( Cheng *et al.*, 2021) with the --primary option. Haplotypic duplications were identified and removed using purge_dups ( Guan *et al.*, 2020). The Hi-C reads ( Rao *et al.*, 2014) were mapped to the primary contigs using bwa-mem2 ( Vasimuddin *et al.*, 2019), and the contigs were scaffolded using YaHS ( Zhou *et al.*, 2023) using the --break option for handling potential misassemblies. The scaffolded assemblies were evaluated using Gfastats ( Formenti *et al.*, 2022), BUSCO ( Manni *et al.*, 2021) and MERQURY.FK ( Rhie *et al.*, 2020).

The mitochondrial genome was assembled using MitoHiFi ( Uliano-Silva *et al.*, 2023), which runs MitoFinder ( Allio *et al.*, 2020) and uses these annotations to select the final mitochondrial contig and to ensure the general quality of the sequence.

***Assembly curation***

The assembly was decontaminated using the Assembly Screen for Cobionts and Contaminants (ASCC) pipeline. Flat files and maps used in curation were generated via the TreeVal pipeline ( Pointon *et al.*, 2023). Manual curation was conducted primarily in PretextView ( Harry, 2022) and HiGlass ( Kerpedjiev *et al.*, 2018), with additional insights provided by JBrowse2 ( Diesh *et al.*, 2023). Scaffolds were visually inspected and corrected as described by Howe *et al*. (2021). Any identified contamination, missed joins, and mis-joins were amended, and duplicate sequences were tagged and removed. Sex chromosomes were identified by synteny analysis. The curation process is documented at https://gitlab.com/wtsi-grit/rapid-curation.

***Assembly quality assessment***

The Merqury.FK tool ( Rhie *et al.*, 2020), run in a Singularity container ( Kurtzer *et al.*, 2017), was used to evaluate *k*-mer completeness and assembly quality for the primary and alternate haplotypes using the *k*-mer databases ( *k* = 31) computed prior to genome assembly. The analysis outputs included assembly QV scores and completeness statistics.

The genome was analysed in the blobtoolkit pipeline, a Nextflow ( Di Tommaso *et al.*, 2017) port of the previous Snakemake Blobtoolkit pipeline ( Challis *et al.*, 2020). It aligns the PacBio reads in SAMtools ( Danecek *et al.*, 2021) and minimap2 ( Li, 2018) and generates coverage tracks for regions of fixed size. In parallel, it queries the GoaT database ( Challis *et al.*, 2023) to identify all matching BUSCO lineages to run BUSCO ( Manni *et al.*, 2021). For the three domain-level BUSCO lineages, the pipeline aligns the BUSCO genes to the UniProt Reference Proteomes database ( Bateman *et al.*, 2023) with DIAMOND blastp ( Buchfink *et al.*, 2021). The genome is also divided into chunks according to the density of the BUSCO genes from the closest taxonomic lineage, and each chunk is aligned to the UniProt Reference Proteomes database using DIAMOND blastx. Genome sequences without a hit are chunked using seqtk and aligned to the NT database with blastn ( Altschul *et al.*, 1990). The blobtools suite combines all these outputs into a blobdir for visualisation.

The blobtoolkit pipeline was developed using nf-core tooling ( Ewels *et al.*, 2020) and MultiQC ( Ewels *et al.*, 2016), relying on the Conda package manager, the Bioconda initiative ( Grüning *et al.*, 2018), the Biocontainers infrastructure ( da Veiga Leprevost *et al.*, 2017), as well as the Docker ( Merkel, 2014) and Singularity ( Kurtzer *et al.*, 2017) containerisation solutions.

Table 4 contains a list of relevant software tool versions and sources.

**Table 4.** Software tools: versions and sources.

Software tool	Version	Source
BLAST	2.14.0	ftp://ftp.ncbi.nlm.nih.gov/blast/executables/blast+/
BlobToolKit	4.3.9	https://github.com/blobtoolkit/blobtoolkit
BUSCO	5.5.0	https://gitlab.com/ezlab/busco
bwa-mem2	2.2.1	https://github.com/bwa-mem2/bwa-mem2
DIAMOND	2.1.8	https://github.com/bbuchfink/diamond
fasta_windows	0.2.4	https://github.com/tolkit/fasta_windows
FastK	666652151335353eef2fcd58880bcef5bc2928e1	https://github.com/thegenemyers/FASTK
Gfastats	1.3.6	https://github.com/vgl-hub/gfastats
GoaT CLI	0.2.5	https://github.com/genomehubs/goat-cli
Hifiasm	0.19.5-r587	https://github.com/chhylp123/hifiasm
HiGlass	44086069ee7d4d3f6f3f0012569789ec138f42b84a a44357826c0b6753eb28de	https://github.com/higlass/higlass
MerquryFK	d00d98157618f4e8d1a9190026b19b471055b22e	https://github.com/thegenemyers/MERQURY.FK
Minimap2	2.24-r1122	https://github.com/lh3/minimap2
MitoHiFi	3	https://github.com/marcelauliano/MitoHiFi
MultiQC	1.14, 1.17, and 1.18	https://github.com/MultiQC/MultiQC
Nextflow	23.04.1	https://github.com/nextflow-io/nextflow
PretextView	0.2.5	https://github.com/sanger-tol/PretextView
purge_dups	1.2.5	https://github.com/dfguan/purge_dups
samtools	1.19.2	https://github.com/samtools/samtools
sanger-tol/ascc	-	https://github.com/sanger-tol/ascc
sanger-tol/blobtoolkit pipeline	0.4.0	https://github.com/sanger-tol/blobtoolkit
Seqtk	1.3	https://github.com/lh3/seqtk
Singularity	3.9.0	https://github.com/sylabs/singularity
TreeVal	1.2.0	https://github.com/sanger-tol/treeval
YaHS	1.2a.2	https://github.com/c-zhou/yahs

### Wellcome Sanger Institute – Legal and Governance

The materials that have contributed to this genome note have been supplied by a Darwin Tree of Life Partner. The submission of materials by a Darwin Tree of Life Partner is subject to the **‘Darwin Tree of Life Project Sampling Code of Practice’**, which can be found in full on the Darwin Tree of Life website here. By agreeing with and signing up to the Sampling Code of Practice, the Darwin Tree of Life Partner agrees they will meet the legal and ethical requirements and standards set out within this document in respect of all samples acquired for, and supplied to, the Darwin Tree of Life Project.

Further, the Wellcome Sanger Institute employs a process whereby due diligence is carried out proportionate to the nature of the materials themselves, and the circumstances under which they have been/are to be collected and provided for use. The purpose of this is to address and mitigate any potential legal and/or ethical implications of receipt and use of the materials as part of the research project, and to ensure that in doing so we align with best practice wherever possible. The overarching areas of consideration are:

•   Ethical review of provenance and sourcing of the material

•   Legality of collection, transfer and use (national and international)

Each transfer of samples is further undertaken according to a Research Collaboration Agreement or Material Transfer Agreement entered into by the Darwin Tree of Life Partner, Genome Research Limited (operating as the Wellcome Sanger Institute), and in some circumstances other Darwin Tree of Life collaborators.

## Data Availability

European Nucleotide Archive: Aethes cnicana (thistle conch). Accession number PRJEB68256; https://identifiers.org/ena.embl/PRJEB68256. The genome sequence is released openly for reuse. The *Aethes cnicana* genome sequencing initiative is part of the Darwin Tree of Life Project (PRJEB40665), the Sanger Institute Tree of Life Programme (PRJEB43745) and Project Psyche (PRJEB71705). All raw sequence data and the assembly have been deposited in INSDC databases. The genome will be annotated using available RNA-Seq data and presented through the Ensembl pipeline at the European Bioinformatics Institute. Raw data and assembly accession identifiers are reported in Table 1 and Table 2.

## Author information

Members of the Wellcome Sanger Institute Tree of Life Management, Samples and Laboratory team are listed here: https://doi.org/10.5281/zenodo.12162482.

Members of Wellcome Sanger Institute Scientific Operations: Sequencing Operations are listed here: https://doi.org/10.5281/zenodo.12165051.

Members of the Wellcome Sanger Institute Tree of Life Core Informatics team are listed here: https://doi.org/10.5281/zenodo.12160324.

Members of the Tree of Life Core Informatics collective are listed here: https://doi.org/10.5281/zenodo.12205391.

Members of the Darwin Tree of Life Consortium are listed here: https://doi.org/10.5281/zenodo.4783558.
