# Supplementary material for: The genome sequence of the Thistle Conch moth, Aethes cnicana (Westwood, 1854)
Source: Wellcome Open Res. 2025 Jun 2;10:289. [Version 1] doi: 10.12688/wellcomeopenres.24252.1 (PMC12198730; doi:10.12688/wellcomeopenres.24252.1)
Supplement: Peer review history

# Reviewer report: Reviewer response for version 1

**DOI:** 10.21956/wellcomeopenres.26749.r124828

**Published:** 2025-06-25

**Authors:** Annabel Whibley

**Affiliations:** 1 Grapevine Improvement, Bragato Research Institute, Lincoln, New Zealand; 2 The University of Auckland, Auckland, Auckland, New Zealand

Young and colleagues report the genome assembly of the tortrid moth *Aethes cnicana* (known as the thistle conch moth). No annotation of the assembly is reported in this Data Note. The report follows Darwin Tree of Life project pipelines, metadata standards and reporting templates.  As such, methods are appropriate, clearly documented and public accession links are active. There is no BOLD confirmation of specimen ID, which would have been a welcome inclusion.

 The authors include an excellent quality photo of the species. These data notes also often include an image of the sequenced specimen and I would have liked to see this included too.

 While the assembly will be a foundational genomic resource for the study of this species, the quality of the assembly falls a little below the habitual DToL Lepidopteran metrics that I see.  I assume that this relates to some features of the sequencing libraries (perhaps read length distribution as coverage would appear ample)  rather than the biology of the genome itself. More specifically, the assembly is relatively fragmented, required many manual curation interventions and has a BUSCO completeness of ~90% when >95% is routinely obtained. The haploid genome size recovered was ~412Mb, considerably lower than the haploid GenomeScope2 estimate of ~340Mb- it would be interesting to unpick this. Also, please specify “GenomeScope2”  in the text of the Sequencing Data section (rather than just “GenomeScope”).

Are sufficient details of methods and materials provided to allow replication by others?

Yes

Is the rationale for creating the dataset(s) clearly described?

Yes

Are the datasets clearly presented in a useable and accessible format?

Yes

Are the protocols appropriate and is the work technically sound?

Yes

Reviewer Expertise:

Genomics, Evolution, Bioinformatics

I confirm that I have read this submission and believe that I have an appropriate level of expertise to confirm that it is of an acceptable scientific standard.

# Reviewer report: Reviewer response for version 1

**DOI:** 10.21956/wellcomeopenres.26749.r125465

**Published:** 2025-06-17

**Authors:** Kay Lucek

**Affiliations:** 1 University of Neuchâtel, Neuchâtel, Switzerland

The authors present the chromosome level genome assembly of a male specimen of the Thistle Conch moth *Aethes cnicana*. The assembly consists of 30 chromosomes including the Z and is not annotated for genes. The assembly is highly complete but interestingly the BUSCO scores seem lower than for other DToL Lepidoptera genomes. The assembly is not phased and I noted two weird clusters on the Hi-C map (Fig. 5) that seem to interact with the rest of the genome. Could this be an assembly error?

 The assembly has been generated using the established pipelines of the Darwin Tree of Life Consortium.

 The presented assembly will be of great value to study the evolution of Lepidoptera. The biology of the species is nicely presented, adding to the value of the genome note.

Are sufficient details of methods and materials provided to allow replication by others?

Yes

Is the rationale for creating the dataset(s) clearly described?

Yes

Are the datasets clearly presented in a useable and accessible format?

Yes

Are the protocols appropriate and is the work technically sound?

Partly

Reviewer Expertise:

Speciation genomics

I confirm that I have read this submission and believe that I have an appropriate level of expertise to confirm that it is of an acceptable scientific standard.
